# Effectiveness and Safety of Complete Decongestive Therapy of Phase I: A Lymphedema Treatment Study in the Greek Population

**DOI:** 10.7759/cureus.9264

**Published:** 2020-07-19

**Authors:** Emmanouil Michopoulos, George Papathanasiou, Georgios Vasilopoulos, Maria Polikandrioti, Evangelos Dimakakos

**Affiliations:** 1 Physiotherapy, University of West Attica, Athens, GRC; 2 Laboratory of Neuromuscular and Cardiovascular Study of Motion, University of West Attica, Athens, GRC; 3 Nursing, University of West Attica, Athens, GRC; 4 Vascular Unit, 3rd Internal Medicine, University of Athens, Sotiria Hospital, Athens, GRC

**Keywords:** physical therapy, treatment, lymphedema, phase i, cdt, greece

## Abstract

Background

Lymphedema is a chronic condition caused by a failure in the lymphatic system that most commonly occurs in the limbs. Complete decongestive therapy (CDT) is the gold standard for lymphedema management.

Objective

To evaluate the effectiveness and safety of complete decongestive therapy (CDT) of phase I in the Greek population with lymphedema.

Methods

The patients’ demographic and clinical characteristics were recorded. CDT was implemented in all patients for 20 sessions in a four-week treatment period. The edema’s (excess volume (EV) and percent of excess volume (PEV)) measurements were carried out four times in the treatment period, whereas the percent reduction of excess volume (PREV) was calculated at the end of phase I. Moreover, we recorded every infection, trauma of skin, and pain of limb during the treatment.

Results

One-hundred five patients with lymphedema were enrolled in the present study, of whom 31.4% had upper limb lymphedema and 68.6% had lower limb lymphedema. All patients with upper limb lymphedema had a secondary type while the corresponding proportion of patients with lower limb lymphedema was 58.3%. A significant reduction (p<0.001) between the pre-treatment and post-treatment values of EV and PEV was found for both upper and lower limb lymphedema. For patients with upper limb lymphedema, the average PREV was 66.5% (interquartile range, 57.3%-80.6%), whereas for patients with lower limb lymphedema, a 71.5% (interquartile range, 64.5%-80.7%) median value was measured. No side effects from the treatment were recorded during CDT.

Conclusion

The proper treatment of the CDT phase I ensures safety and a great reduction in edema in patients with lymphedema that predispose the success of phase II of CDT.

## Introduction

The lymphatic system consists of vessels that collect and drain lymph fluid from the skin, tissue, muscle, and bone. After draining the fluid, the lymphatic system will return it to circulation [1]. In addition to its role in the maintenance of tissue ﬂuid homeostasis, the lymphatic system participates actively in lipid absorption and transport from the gastrointestinal lumen and in the trafﬁc of immunocompetent cells from the periphery to the central lymphoid tissues. Lymphedema occurs when there is some sort of disruption to one or more of these functions, and it can be either heritable or an acquired form [2]. This insufficiency of the lymphatic system causes an accumulation of protein-rich interstitial fluid, leading to distention, the proliferation of fatty tissue, and progressive fibrosis [3].

Approximately 80% of lymphatic drainage must be nonfunctional before lymphedema becomes clinically evident [4]. It is more commonly noted in the limbs, but it can also affect the head, neck, breast, or genitalia [3]. It is estimated that approximately 140 million to 250 million people worldwide have lymphedema [4]. There are many different causes of this condition, which fall into two categories: primary lymphedema and secondary lymphedema [5]. Cancer treatment (especially breast cancer treatment), malignancy, nematode infection (filariasis), and trauma are responsible for >90% of cases worldwide. In developed countries, like Greece, iatrogenic injury from the treatment of breast cancer is the most common cause [4]. Six-month prevalence rates, for any six months within a three-year window, have been estimated at 23% to 29% [6]. It is reported that women with breast cancer-related lymphedema (BCRL) show medical costs $14,887 to $23,167 higher than women with breast cancer without BCRL. Indirect costs (e.g., workdays lost) are also higher for BCRL [7]. This opinion that chronic lymphedema is associated with high direct and indirect costs is also supported by other studies [8-9].

Complete or complex decongestive therapy (CDT), also known as complex decongestive physiotherapy (CDP) [10], is currently recognized as one of the treatments of lymphedema [11-13]. Also, the term "decongestive lymphatic therapy (DLT)" is often used as a suitable name for this group of therapeutic techniques [14]. Usually, the treatment of lymphedema has followed a "two-phase" approach: phase Ι or intensive phase (decongestion phase) and phase II or maintenance phase [15]. The aim of phase I is to mobilize and to reduce the congested protein-enriched fluid and, if present, to soften and reduce the increased connective tissue. The objective of phase II is to optimize and preserve the results already achieved at the end of phase I [16].

The purpose of this study was to explore the effectiveness and safety of phase I (intensive phase) CDT among Greek patients with lymphedema in their limbs.

## Materials and methods

Study population

One-hundred five patients were enrolled in the present study. Although 144 patients were indicted for treatment, 39 subjects were excluded from the study, as they failed to meet the entry and output criteria. This prospective study took place from March 2017 to April 2019 and was conducted in the Laboratory of Neuromuscular and Cardiovascular Study of Motion (LANECASM) of the University of West Attica and in the Vascular Unit of 3rd Internal Medicine of the University of Athens of Hospital “Sotiria.”

Patients were assessed for eligibility by a physician and physiotherapist specialized in the field of lymphology. A full medical evaluation, as well as exploratory examinations, was carried out to ensure the diagnosis of lymphedema. These tests, depending on the patient's condition, included blood tests, a duplex colored vein of the upper or lower limb, lymphoscintigraphy, and computed tomography (CT).

Criteria for the inclusion of patients in the study were: (1) unilateral lymphedematous limb, (2) absence of a history of previous treatment of the lymphedema of the affected side, (3) absence of absolute contraindication for the application of CDT intervention, and (4) absence of any neuromuscular disease that can be related to the affected limb.

The exclusion criteria for patients were as follows: (1) with severe heart disease, (2) with uncontrolled hypertension, (3) with active lymphangitis, (4) with venous thrombosis, and (5) those who did not fully attend the therapy (five times per week for four weeks).

The study was approved by the Thesis Review Committee of the Institute, and it was conducted in accordance with the Declaration of Helsinki (1989) of the World Medical Association. All patients participated in the study voluntarily and had their anonymity preserved. Written informed consent was obtained from all participants.

Data variables

The data collected for each patient included the following: sex (male or female), age, type of lymphedema (primary or secondary), disease staging (I or II or III), duration of lymphedema, baseline total volume in the affected and unaffected limb, edema (excess volume (EV) and percent of excess volume (PEV)) and percent reduction of excess volume (PREV). Moreover, every infection or trauma of skin or pain of the limb during the treatment were recorded.

Lymphedema in patients was classified as primary or secondary depending on the cause of the damage or disruption of lymph function. After determining the type of lymphedema, the stage of lymphedema was defined according to the instructions of the International Society of Lymphology (ISL), which uses a specific scale for the classification of a lymphedematous limb (0-III) [12].

Validated automated software (Limb Volume Professional, Bioscience Research Institute) was used to calculate the baseline total volume in the affected and unaffected limb, the excess volume (EV), and the percent of excess volume (PEV). In order for the software to be able to calculate the above variables, the values of circumferences of both limbs of each patient were recorded, but first, the length of each limb was divided into 4 cm intervals. The limb was marked at the chosen segment lengths starting at the wrist and ankle for the upper and lower limb, respectively. Measure circumferences at each mark are also including the final length mark, which is the axillary crease for the upper limb and the groin crease for the lower limb.

Finally, for each patient of the study, to determine the efficacy of CDT, the response to the therapeutic intervention, was calculated the percent reduction of excess volume (PREV). This percentage was calculated as follows: (pre-treatment EV - post-treatment EV)/pretreatment EV × 100%, which has been used in several related studies [17-19].

CDT was performed in all patients for 20 sessions in a four-week treatment period. During the study, the EV and PEV variables were measured four times at equal intervals for all patients. These measurements were carried out by an independent evaluator physician with the first measurement taking place in the first session and the fourth in the last (twentieth session). The PREV variable was calculated at the end of phase I (at four weeks).

Phase I CDT (intensive phase)

In all patients, phase I CDT was applied. This phase consists of the following components: manual lymphatic drainage (MLD), compression therapy, specific remedial exercises, and skincare [11]. In this study, treatment was performed daily (five times/week), one session per day, except for weekends, for a period of four weeks (20 sessions). Each session consisted of (1) 60 minutes of MLD according to Vodder’s techniques, (2) compression, which was applied after MLD by multilayered, short-stretch bandages, which were worn in between sessions and reapplied at each session, (3) specific exercises that were performed with a bandage in place to enhance lymphatic flow, and (4) meticulous skincare before applying the bandage. The therapeutic protocol was performed by a physiotherapist specialized in the treatment of lymphedema.

Statistical analysis

Using the Kolmogorov-Smirnov criteria, the distributions of the quantitative variables were checked for the regularity of their distribution. Mean values and standard deviations (SD) were used for those which were normally distributed, while median and interquartile ranges (IQR) were additionally used for those that were not normally distributed. Absolute (N) and relative (%) frequencies were used to describe the qualitative variables. The non-parametric Mann-Whitney criteria were used to compare quantitative variables between the two groups. Repeated measures of analysis of variance (ANOVA) were used to test for differences in measurements over time. Scatter analysis for repeated measurements was performed using logarithmic transforms. All tests were two-sided at the 0.05 signiﬁcance level and analyses were carried out using the statistical program SPSS version 22.0 (IBM Corp., Armonk, NY).

## Results

Descriptives

A total of 105 patients was enrolled in the study. Table 1 presents the baseline demographic and clinical characteristics. Among these patients, 33 patients (31.4%) had upper limb lymphedema and 72 patients (68.6%) had lower limb lymphedema. All patients with upper limb lymphedema were women while the corresponding proportion of patients with lower limb lymphedema was significantly lower and equal to 70.8%. Furthermore, all patients with upper limb lymphedema had the secondary type while the corresponding proportion of patients with lower limb lymphedema was significantly lower and equal to 58.3%.

**Table 1:** Baseline demographic and clinical characteristics EV: excess volume; PEV: percent of excess volume; SD: standard deviation; IQR: interquartile range

Characteristics	Upper Limb (N=33, 31.4%)			Lower Limb (N=72, 68.6%)		
	N (%)	Mean (± SD)	Median (IQR)	N (%)	Mean (± SD)	Median (IQR)
Sex						
Males	0 (0)			21 (29.2)		
Females	33 (100)			51 (70.8)		
Age (years)		55.3 (± 10.3)			56.4 (± 12.0)	
Type of lymphedema						
Primary	0 (0)			30 (41.7)		
Secondary	33 (100)			42 (58.3)		
Disease staging						
I	3 (9.1)			3 (4.2)		
II	24 (72.7)			51 (70.8)		
III	6 (18.2)			18 (25)		
Duration of lymphedema						
≤ 1 year	12 (36.4)			36 (50)		
> 1 year	21 (63.6)			36 (50)		
Baseline total volume in the affected limb (ml)		5107.5 (± 2850.9)	4204.0 (3765.0–6179.0)		13417.3 (± 4878.3)	12363.5 (9979.5–13847.5)
Baseline total volume in the unaffected limb (ml)		3191.5 (± 1043.8)	2942.0 (2653.0–3591.0)		8924.3 (± 3112.8)	8947.0 (7266.0–10099.0)
Baseline edema						
EV (ml)		1916.1 (± 1972.4)	1262.5 (784.6–2072.5)		4493.0 (± 3435.8)	3219.1 (2236.8–5569.9)
PEV (%)		54.7 (± 41.4)	42.9 (27.9–58.2)		54.6 (± 42.8)	33.2 (26

The majority of lymphedema was Stage 2. More specifically, concerning the lymphedema in the upper limb, 9.1% was stage 1, 72.7% stage 2, and 18.2% stage 3. Respectively, about the lymphedema in the lower limb, 4.2% was stage 1, 70.8% stage 2, and 25% stage 3. As regards the duration of lymphedema, 63.6% of patients with upper limb lymphedema were more than 1 year and the corresponding proportion of patients with lower limb lymphedema was 50%.

The severity of lymphedema was classified according to the instructions of the International Society of Lymphology (ISL) [11]. The median value of PEV in the upper and lower limbs, prior to treatment, was 42.9% (interquartile range, 27.9% -58.2%) and 33.2% (interquartile range, 26.1% -82.6%), respectively. Therefore, according to the ISL classification, patients with upper limb lymphedema had marginal severe lymphedema while patients with lower limb lymphedema had moderate lymphedema.

Responses to phase I CDT

**Figure 1:**
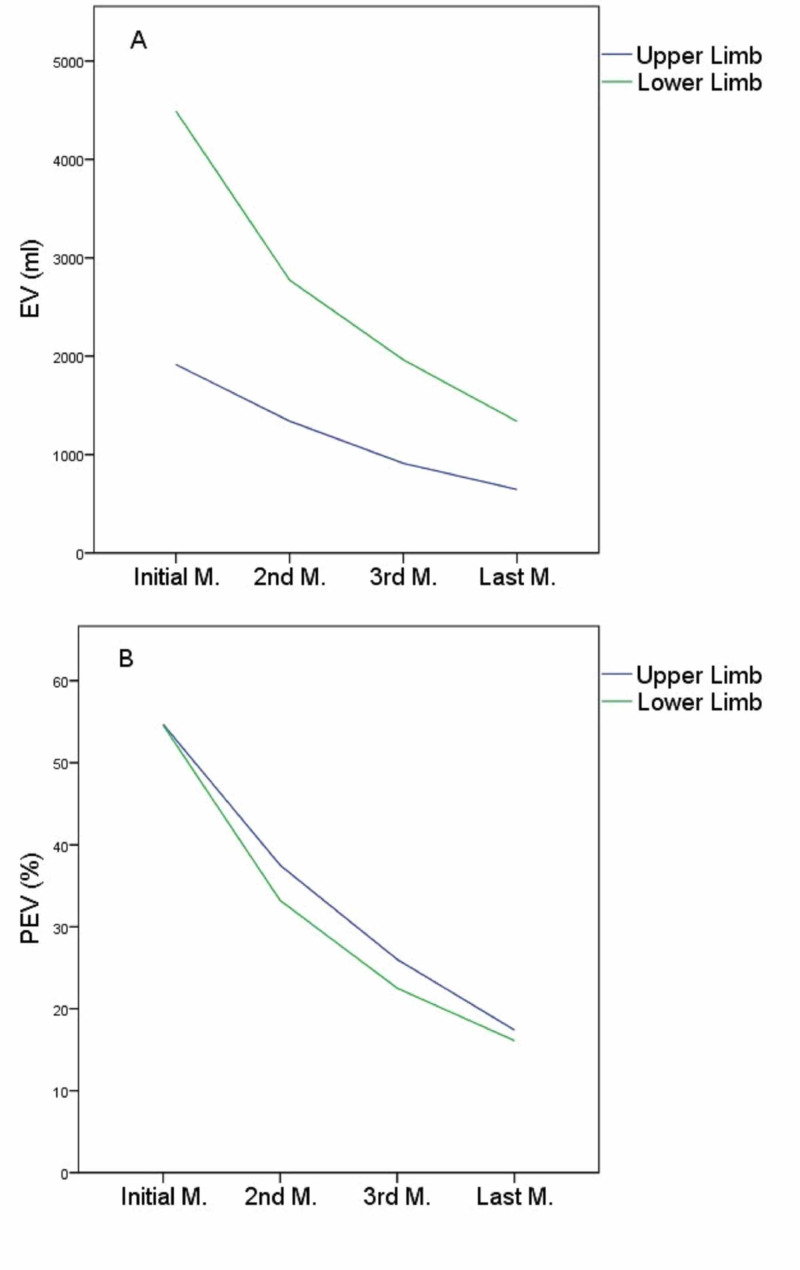
(a) Distribution of EV at study time measurements in patients with lymphedema in the upper or lower limb. (b) Distribution of PEV at study time measurements in patients with lymphedema in the upper or lower limb Both (a) and (b) figures describe the mean values of EV and PEV in time monitoring, respectively. Initial measurement is the first-baseline measurement (pre-treatment value) and last measurement is the fourth measurement (post-treatment value). EV: excess volume; PEV: percent of excess volume; M: measurement

**Figure 2:**
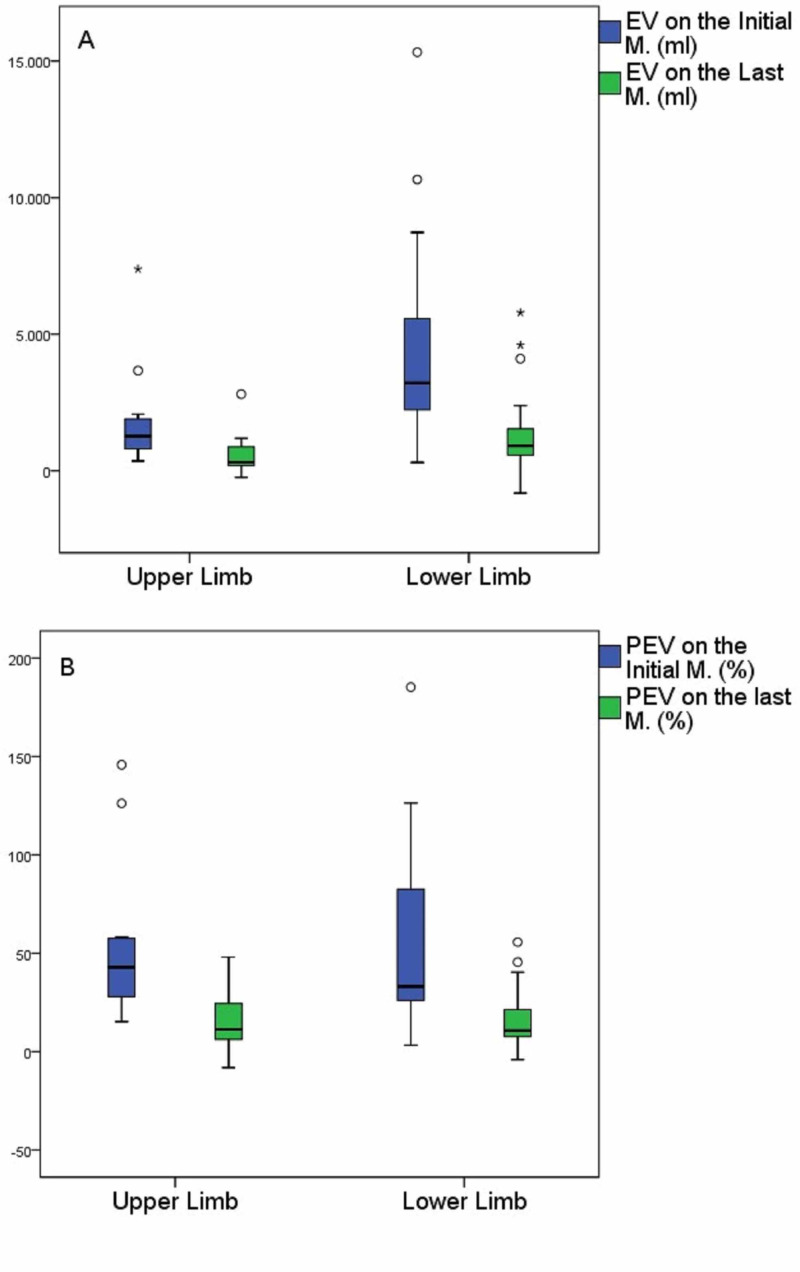
(a) Comparison of EV between initial and last measurements in patients with lymphedema in the upper or lower limb. (b) Comparison of PEV between Initial and Last measurement in patients with lymphedema in upper or lower limb Initial measurement is the first-baseline measurement (pre-treatment value) and last measurement is the fourth measurement (post-treatment value). Horizontal bar in the box: median; Circles: outliers; Asterisks: extreme points; Box: interquartile range (IQR); Bars: 1.5×IQR EV: excess volume; PEV: percent of excess volume; M.: measurement

**Figure 3:**
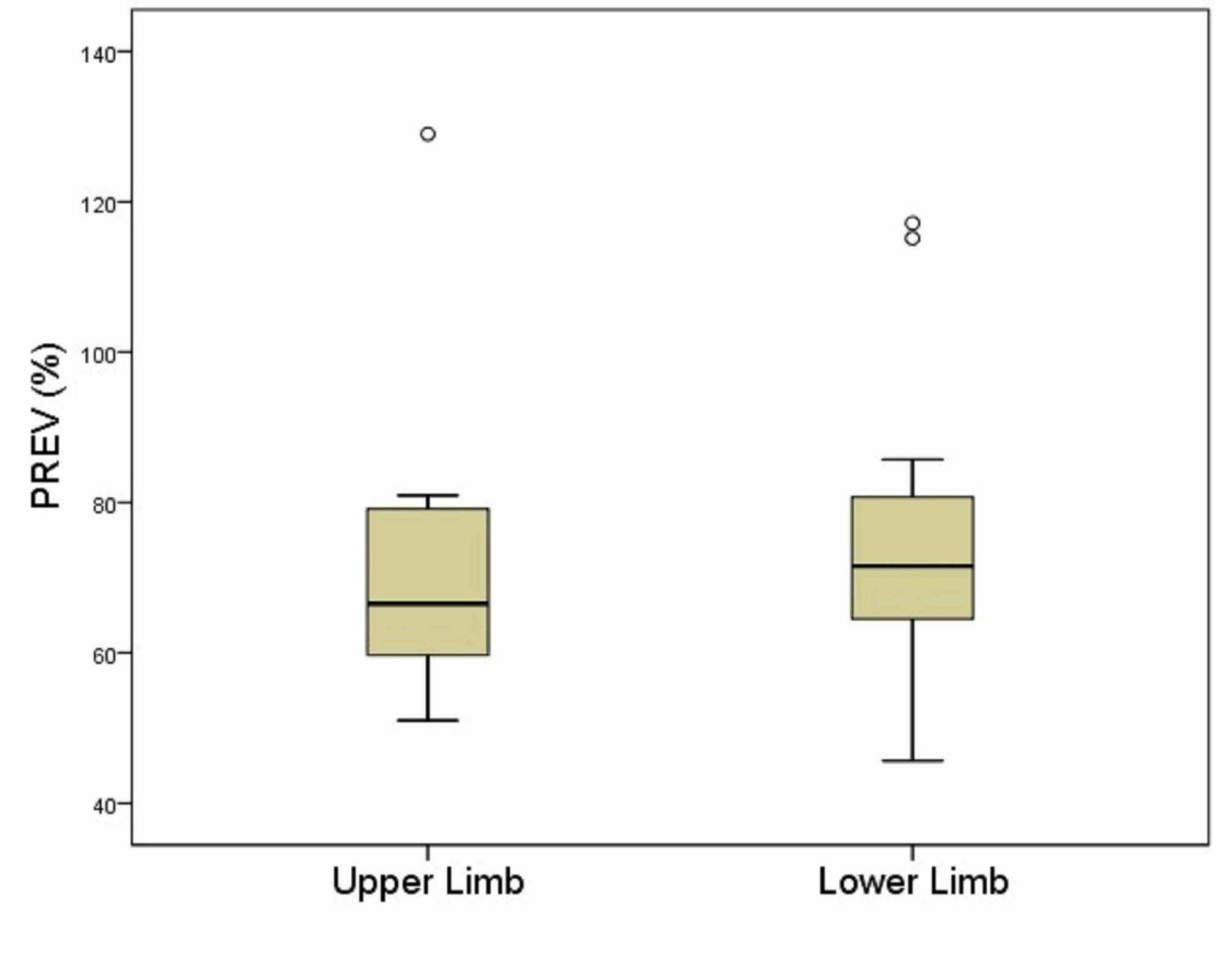
Τhe efficacy of CDT (PREV) in patients with lymphedema in the upper or lower limb Horizontal bar in the box: median; Circles: outliers; Box: interquartile range (IQR); Bars: 1.5×IQR PREV: percent reduction of excess volume

The responses to phase I CDT are summarized in Table 2 and Figures 1-3. More specifically, Table 2 presents the change of the edema and the efficacy of CDT (PREV) in patients with lymphedema in the upper or lower limb. Both variables (EV and PEV) that constitute the edema significantly decreased in follow-up time in both patient groups (p <0.001).

**Table 2:** Change in edema and the efficacy of CDT (PREV) in patients with lymphedema in the upper or lower limb The initial measurement is the first-baseline measurement (pre-treatment value) The last measurement is the fourth measurement (post-treatment value) * ANOVA test for time effect (using logarithmic transforms) ** comparison of the PREV between patients with lymphedema in the upper and the lower limb (Mann-Whitney test) ***p<0.05 CDT: complete decongestive therapy; PREV: percent reduction of excess volume; EV: excess volume; PEV: percent of excess volume; SD: standard deviation; IQR: interquartile range; ANOVA: analysis of variance

	Upper Limb		Lower Limb	
	Mean (± SD)	Median (IQR)	Mean (± SD)	Median (IQR)
Edema on the initial measurement				
EV (ml)	1916.1 (± 1972.4)	1262,5 (784,6─2072.5)	4493.0 (± 3435.8)	3219.1 (2236.8─5569.9)
PEV (%)	54.7 (± 41.4)	42.9 (27.9─58.2)	54.6 (± 42.8)	33.2 (26.1─82.6)
Edema on the 2^nd^ measurement				
EV (ml)	1340.2 (± 1493.9)	750.0 (426.0─1617.0)	2774.8 (± 2533.5)	1906.5 (1335.5─3209.0)
PEV (%)	37.5 (± 32.0)	26.9 (14.4─39.3)	33.2 (± 28.0)	21.2 (15.1─47.3)
Edema on the 3^rd^ measurement				
EV (ml)	912.2 (± 1015.7)	481.1 (253.1─1209.2)	1964.2 (± 1909.3)	1425.9 (866.6─2267.5)
PEV (%)	26.0 (± 25.3)	16.4 (7.5─33.0)	22.5 (± 21.2)	15.3 (11.2─32.5)
Edema on the last measurement				
EV (ml)	646.0 (± 804.5)	315.0 (175.8─1014.8)	1338.5 (± 1488.2)	916.6 (577.1─1539.7)
PEV (%)	17.4 (± 17.1)	11.3 (5.8─24.7)	16,1 (± 15,1)	10.8 (7.6─21.5)
p-value*	<0.001***	<0.001***	<0.001***	<0.001***
PREV	71.9 (± 20.7)	66.5 (57.3-80.6)	73.6 (± 16.5)	71.5 (64.5-80.7)
p-value**	0.106			

For patients with upper limb lymphedema, the variable EV decreased significantly from the initial measurement with a median of 1262.5 ml (interquartile range, 784.6-2072.5 ml) to the second measurement with a median of 750 ml (interquartile range, 426-1617ml; p<0.001). In the third measurement, it also decreased significantly with a median of 481.1 ml (interquartile range, 253.1-1209.2 ml; p=0.018), and in the final measurement, it decreased significantly further with a median of 315 ml (interquartile range, 175.8-1014.8 ml; P=0.001). Concerning the PEV variable, it decreased significantly from the initial measurement with a median of 42.9% (interquartile range, 27.9%-58.2%) to the second measurement with a median of 26.9% (interquartile range, 14.4%-39.3%; p<0.001). In the third measurement, it did not change significantly with a median value of 16.4% (interquartile range, 7.5%-33%; p>0.05), but in the last measurement, it showed a significant decrease with a median value of 11.3% (interquartile range, 5.8%-24.7%; p<0.001).

For patients with lower limb lymphedema, the EV variable was also significantly reduced from initial measurement with a median of 3219.1 ml (interquartile range, 2236.8-5569.9 ml) to the second measurement with a median of 1906.5 ml (interquartile range, 1335.5-3209; p<0.001). At the subsequent measurements, it remained at similar levels, with median values for the third and last measurement 1425.9 ml (interquartile range, 866.6-2267.5 ml; p>0.05) and 916.6 ml (interquartile range, 577.1-1539.7 ml; P>0.05), respectively. Concerning the PEV variable, it decreased significantly from the initial measurement, with a median of 33.2% (interquartile range, 26.1%-82.6%) to the second measurement with a median of 21.2% (interquartile range, 15.1%-47.3%; p<0.001). In the third measurement, it did not change significantly, with a median value of 15.3% (interquartile range, 11.2%-32.5%; p>0.05), but in the last measurement, it showed a significant decrease, with a median value of 10.8% (interquartile range, 7.6%-21.5%; p=0.002).

The above changes in the EV and PEV variables are depicted in Figure 1a and Figure 1b, respectively. In addition, comparisons between the initial and last measurements for the EV and PEV variables are attributed to Figure 2a and Figure 2b, respectively.

Finally, the degree of reduction of edema is described in Table 2 via the variable PREV and is depicted in Figure 3. This rate was similar for both categories of patients, with a median value of 66.5% (interquartile range, 57.3%-80.6%) for patients with upper limb lymphedema and 71.5% (interquartile range, 64.5%-80.7%) for those with lower limb lymphedema.

Concerning the safety of CDT in phase I, we did not record any infection, trauma, or pain during the treatment. Moreover, we did not record complaints from the patients concerning the treatment of CDT.

## Discussion

Lymphedema is a significant chronic issue not only for the patients themselves but also for the medical community worldwide. The results of the present study have shown that phase I of CDT is an effective therapeutic method that has a great positive effect on lymphedema reduction. The percent of edema reduction in 105 Greek patients for both the upper and lower limbs was quite high (66.5% and 71.5%, respectively) with the duration of treatment being four weeks for all patients (20 sessions). Relevant studies have shown that edema reduction may range from 22% to 78%, with the number of sessions varying from six to 36, however, they are no recent studies [20-23].

In a prior study of 299 patients with upper or lower limb lymphedema, primary and secondary type, where CDT was applied for about 15.7 days, at the end of phase I of CDT, an edema reduction of 59.1% for the upper limb and 67.7% for the lower limb was demonstrated [24]. In a relevant study conducted by Yamamoto, CDT was applied in 82 women for a median duration of six and 10 days for upper or lower limb lymphedema. Results showed a 58.9% edema reduction for the upper limb and 73.4% for the lower limb [25]. Morgan et al. showed a reduction greater than 50% among 78 BCRL patients having grade I and II lymphedema who followed CDT for one month (five days per week) [26].

Szuba, Cooke, Yousuf, and Rockson (2000) applying CDT intervention for a mean duration of 8 ± 3 days in 79 patients with upper or lower limb lymphedema, reported a reduction of edema by 44% and 42%, respectively [18]. But in this prospective study of therapeutic responses in chronic lymphedema, phase I of CDT was combined with patient instruction in self-care (self-massage and self-bandaging). Hammer and Fleming (2007) conducted a study involving phase I CDT in 135 patients with BCRL. The mean initial lymphedema volume was 709 ml and after eight weeks (two sessions per week) of intervention, it was 473 ml and the edema decreased by 41.7%. Phase I of CDT again was combined with instruction in self-care [27].

Even if the greater part of the reduction of the volume of the lymphedema happens in the first five days of treatment, according to Leduc O, Leduc A, Bourgeois, and Belgrado (1998), after that, another reduction will be achieved but it will be less than the first. However, it should be noted that patients in this study were only studied for the first two weeks of treatment [28]. Also, Vignes, Blanchard, Arrault, and Porcher (2013) report that concerning the phase I CDT better results are available at 11 days of treatment than at four, with 63% of the total edema reduction achieved at four days for 39% of patients. Again, in this study, the study time of the population was limited. In our study, the greatest reduction was achieved as expected at the second measurement with the decrease continuing but to a lesser extent until the end of the treatment [29].

There was a tendency for people with a longer duration of lymphedema and a higher degree of fibrosis to receive more sessions. However, Yamamoto R and Yamamoto T, 2007, found that there is no correlation between the duration of lymphedema and the duration of phase I CDT [25]. Furthermore, Liao et al. (2013) state that the reduction of edema is not related to the number of sessions in phase I CDT [30].

The effectiveness of the intervention of CDT of phase I varies in different studies and the wide range in terms of dosage (minutes of the session, number of sessions per week, and number of weeks), which observed among the researchers and the lack of comparison among these studies indicate the need to develop a common protocol of treatment for better provision of services. More research is, therefore, needed to identify the factors affecting the effectiveness of CDT in phase I. Perhaps the use of the same protocol of treatment worldwide could be useful for the therapists and beneficial for the patients.

Concerning the safety of CDT in phase I, we had not any remarkable problem in patients of our study. We did not record any redness, pain, infection, or trauma or any complaint from the lymphedema patients, during the treatment. Prerequisites of successful treatment of CDT are the availability of physicians (i.e., clinical lymphologists), nurses, physiotherapists, occupational, and other therapists specifically trained, educated, and experienced in this method [12]. Any infection or trauma could stop the treatment of CDT. It is important for the therapist to follow the correct treatment of CDT in order to achieve greater success in treatment without local complications.

Limitations of the study

This study has some limitations. The sample is not representative of patients with lymphedema living in Greece. Moreover, perhaps the absence of a control group with a different treatment of CDT could give us more information, although our study treatment showed a great reduction of edema and safety.

## Conclusions

Conclusively, the treatment of lymphedema with CDT phase I showed positive safety and great effectiveness on the treatment of lymphedematous limbs in the population of the present study. The correct treatment of CDT phase I ensure safety and significant reduction of edema in patients with Lymphedema that predispose the success of phase II of CDT.
